# Unravelling the noncanonical extracellular DNA structures in biofilm and NETosis

**DOI:** 10.1093/nar/gkaf1505

**Published:** 2026-01-09

**Authors:** Tevriz Dilan Demir, Satoe Azechi-Ogawa, Nikhil Ram-Mohan, Samuel Yang

**Affiliations:** Department of Emergency Medicine, Stanford University School of Medicine, Palo Alto, CA 94305, United States; Department of Emergency Medicine, Stanford University School of Medicine, Palo Alto, CA 94305, United States; Department of Emergency Medicine, Stanford University School of Medicine, Palo Alto, CA 94305, United States; Department of Emergency Medicine, Stanford University School of Medicine, Palo Alto, CA 94305, United States

## Abstract

Noncanonical secondary structures of DNA have been well characterized *in vitro* for their catalytic and sensory functions, as well as *in vivo* for their regulatory functions in the genome. However, their presence and functional significance in the extracellular DNA (eDNA), particularly within biofilms and neutrophil extracellular traps (NETs), have only recently begun to be appreciated and have yet to be fully understood. Emerging studies have identified these atypical DNA conformations as integral components that contribute to the structural stability of biofilms and antimicrobial activity of NETs. In this personal view, we advocate for a comprehensive investigation of these unconventional DNA structures within extracellular contexts, where their distinct physiochemical properties are exposed to dynamic and unpredictable microenvironments, with the potential to profoundly influence microbial behaviour, immune responses, and host–pathogen interactions. Considering the broad spectrum of diseases associated with biofilm and NETs, targeting noncanonical eDNA structures may offer novel therapeutic avenues and shed light on mechanisms of immune tolerance and dysregulation.

## Graphical Abstract

**Graphical Abstract:**
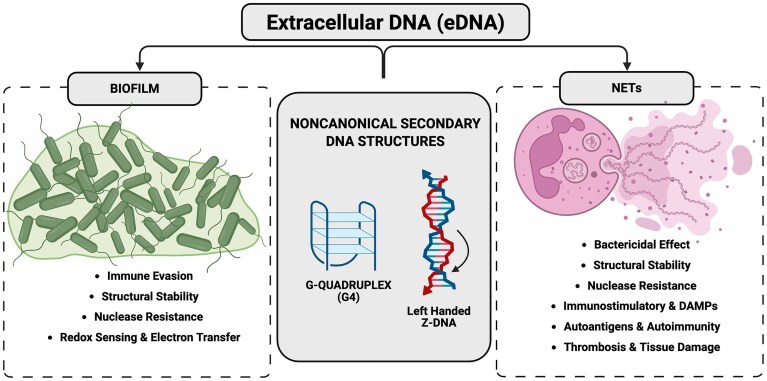

## Introduction

Noncanonical secondary structures of DNA, deviating from the classical B-DNA double helix, have been found in the genomes of thousands of organisms across the tree of life [1–3]. Formation of structures such as G-quadruplexes (G4), Z-DNA, Holliday junctions (HJ), cruciforms, triplexes, hairpins, and i-motifs can be strongly influenced by specific sequence motifs that are widely distributed throughout the genome [4]. For example, G4 arise from guanine-rich sequences where four guanines form Hoogsteen hydrogen bonds, resulting in planar tetrads [5]. In contrast, Z-DNA is in equilibrium with B-DNA, and its formation is not strictly sequence specific and, in principle, can occur at any sequence under sufficient negative supercoiling. Nevertheless, alternating purine-pyrimidine/GC-rich tracts (e.g. poly(dG-dC)) substantially lower the energetic threshold for the B-to-Z transition, often making them ideal genomic sites for Z-DNA formation [6]. Beyond primary sequences, formation of these dynamic alternative conformations also depends on various cellular and environmental factors, such as superhelical stress, chromatin conformation [7], oxidative stress, ligands, ions, and pH [8–10]. Intracellularly, these structures on nuclear DNA have been implicated in the regulation of biological processes such as replication, transcription, recombination, maintaining genetic stability, DNA damage and repair [4], and protecting cells from hemin toxicity [11]. However, little is known about their existence or potential roles in extracellular DNA, particularly the functionalized bioactive free eDNA found in biofilms and neutrophil extracellular traps (NETs)—two biologically distinct yet pathologically convergent systems. Extracellular DNA (eDNA) can arise not only from passive release (e.g. cell lysis or apoptosis) but also from programmed regulated processes such as NETosis, autolysis, or phage-mediated lysis, where DNA release appears to occur in response to stress, infection, or disease [12]. Given the conditions outside a cell differ drastically from those within, while the noncanonical secondary structures primarily regulate gene expression intracellularly, their properties and functions are likely distinct and more diverse in the complex extracellular environments subject to unpredictable perturbations.

**Figure 1.**
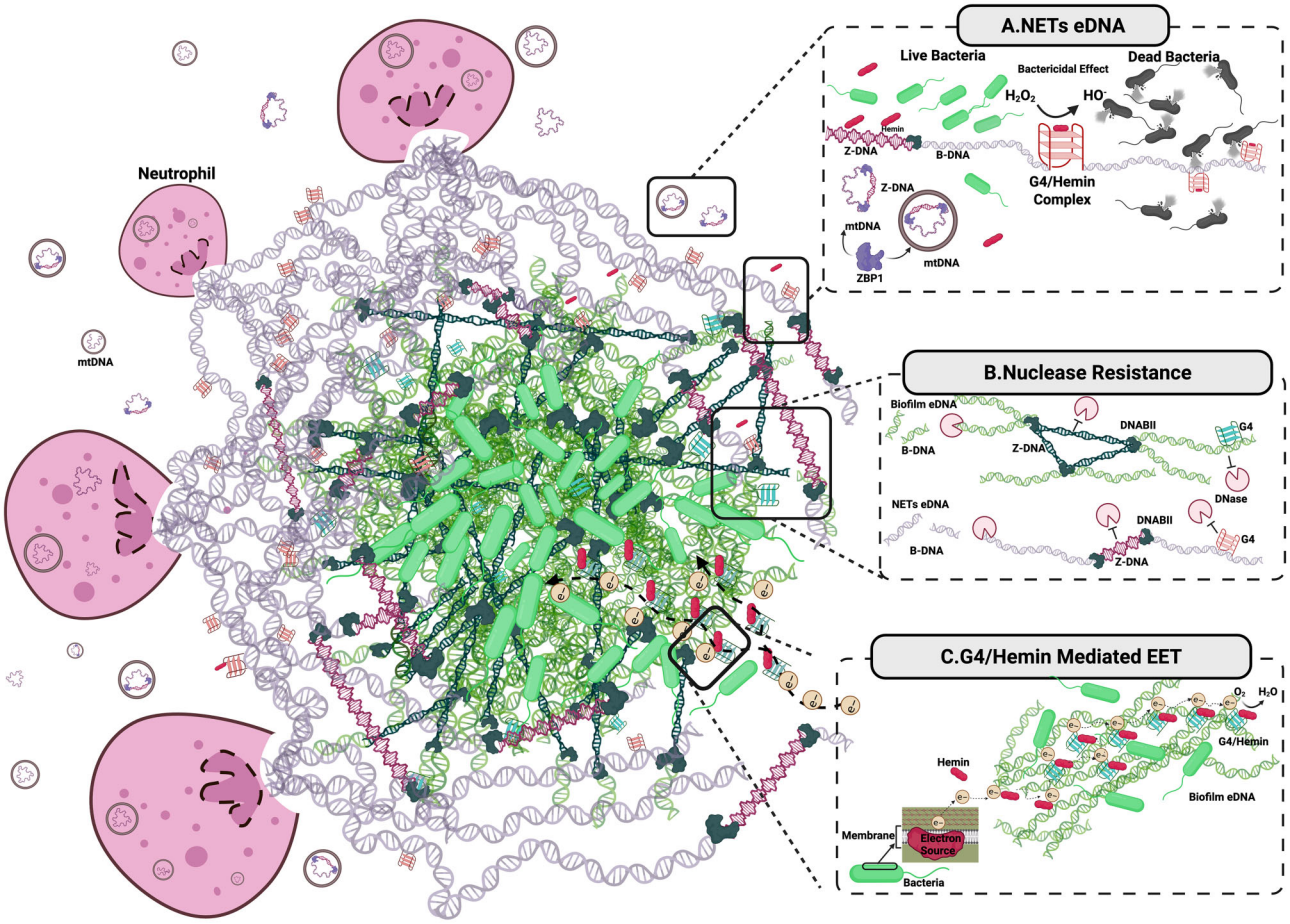
Conceptual model of bioactive eDNA structures and their role in host–pathogen interactions within the context of NETs and bacterial biofilms. This illustration depicts key structural and functional features of eDNA released during NETosis and its interaction with a component of a schematic aggregate bacterial biofilm. NETs entrap bacteria and contain structurally diverse DNA elements, including Z-DNA stabilized by bacterial DNABII proteins, G4, and mitochondrial DNA (mtDNA). Host-derived Z-DNA-binding protein 1 (ZBP1) binds Z-DNA motifs released in both free circular or vesicle-associated mtDNA, stabilizing the left-handed conformation. Within bacterial biofilms, structural integrity is reinforced by both G4 and Z-DNA stabilized through interactions of DNABII proteins at Holliday junctions. (**A**) NETs eDNA: highlights the bactericidal potential of G4/hemin complexes, which exhibit peroxidase activity by converting H₂O₂ into hydroxyl radicals (•OH), leading to bacterial killing. In contrast, unbound hemin alone does not induce bactericidal effects. Nuclear and mitochondrial DNA released during NETosis are subject to conditions shifting the equilibrium towards the formation of Z-DNA in DNase rich blood and have to cast a wide NET to trap pathogens. NETs can adopt Z-form in the presence of bacterial DNABII, and ZBP1 binds to mtDNA. (**B**) Nuclease resistance: illustrates that both G4 and Z-DNA, anchored by Holliday junctions in biofilm, and NET eDNA resist degradation by nucleases such as DNase I and DNase1L3, whereas canonical B-form DNA is susceptible to enzymatic digestion. (**C**) G4/hemin-mediated EET: Presents a conceptual model for G4/hemin-mediated extracellular electron transfer (EET) in biofilms, suggesting that noncanonical eDNA structures participate in redox reactions within the extracellular matrix. Together, these panels propose that noncanonical eDNA structures form a structural and functional interface between host and pathogens, contributing to antimicrobial defence, immune modulation, and biofilm resilience. Created in BioRender. Demir, T. D. (2025) https://BioRender.com/n2fzaih.

Biofilms and NETs represent fascinating biological systems both rich in eDNA [13, 14]; however, the noncanonical structures of eDNA are still underexplored in these contexts. In addition to promoting structural stability in both systems [15–17], these alternative forms may also contribute to immune evasion in biofilm and antimicrobial defence in NETs [16, 17] (Fig. 1A). Moreover, these conformations may have unique immunostimulatory potential, acting as potent structural pathogen-associated molecular patterns (PAMPs) or damage-associated molecular patterns (DAMPs), triggering and amplifying immune responses from pattern-recognition receptors (PRRs) with implications in prolonged inflammation and autoimmune activation. Given the disparate biological settings from which eDNA arises in biofilms or NETs, as well as their dynamic interplay with each other as part of host–pathogen interaction [16], these systems offer a unique opportunity to draw comparisons and gain novel insights into the roles of noncanonical eDNA structures in disease mechanisms, microbial behaviours, and immune responses. In this perspective, we propose that unique secondary structures are integral to eDNA and that studying these less understood systems would uncover new biological roles of eDNA and highlight their potential as therapeutic targets. We will focus on G4 and Z-DNA due to the greater body of evidence supporting their relevance in these contexts compared to other structures.

### Structural basis of noncanonical secondary DNA structures

G4s arise predominantly from guanine-rich sequences, typically following the consensus motif G≥3N1–7G≥3N1–7G≥3N1–7G≥3 [18], in which guanines form planar tetrads through Hoogsteen hydrogen bonding and stack into higher-order structures. They can adopt intramolecular (within a single strand) or intermolecular (between two or more strands) configurations and display diverse topologies—parallel, antiparallel, or hybrid—depending on loop arrangement, strand orientation [19], and syn/anti glycosidic conformations of guanines [20]. A simple rule links strand orientation with glycosidic conformation: guanines aligned in the same strand direction adopt the same glycosidic conformation, whereas guanines in opposite orientations adopt opposite conformations [20]. This structural diversity allows G4s to adapt dynamically to environmental conditions. Their stability is strongly dependent on cations, with potassium conferring the greatest stabilization, sodium supporting antiparallel folding, and divalent ions such as magnesium and calcium modulating stability under physiological conditions [3, 19]. Given that inflamed tissues often exhibit local sodium accumulation and altered ionic microenvironments, and that biofilm growth is supported by increased salt concentrations, these ionic dependencies may critically shape the stability and persistence of extracellular G4s [21–23]. Beyond ionic regulation, naturally occurring small molecules can also act as G4 ligands. Hemin, a heme-derived porphyrin released during hemoprotein turnover and inflammation, binds and stabilizes G4s and forms catalytically active G4/hemin DNAzymes [11, 17, 24]. Likewise, polyamines such as spermine and spermidine—abundant in mammalian cells—interact electrostatically with DNA and contribute to G4 stabilization [25–27]. Together, these endogenous ligands suggest that the cellular milieu provides not only ions but also bioactive metabolites that can modulate G4 conformation and function. In addition to these physiological ligands, a wide spectrum of artificial small molecules, including porphyrin derivatives, acridines, and quinolines, have been designed to selectively stabilize or destabilize G4s, expanding their potential as therapeutic targets [28].

Z-DNA, on the other hand, represents a left-handed helical conformation of the double helix, characterized by a zig-zag phosphate backbone and alternating syn/anti glycosidic conformations [29] that is formed without sequence specificity but with a higher propensity towards alternating purine–pyrimidine motifs [30]. Formation is strongly promoted by base modifications, the presence of Z-alpha domain-containing proteins, negative supercoiling during transcription, mechanical strain, and high salt or polycationic environments [31, 32]. A wide range of ligands, like natural polyamines such as spermine and spermidine, can stabilize Z-DNA like G4 [33]. Unlike G4s, which rely primarily on cation-mediated Hoogsteen stabilization, Z-DNA formation depends on torsional stress and electrostatic neutralization, highlighting a mechanistic contrast between these noncanonical structures [32, 34].

pH is likely a potential regulator of the dynamic equilibrium between the B and noncanonical structures of the DNA. Low pH can favour DNA towards non-B conformations or favour a switch between two non-B conformations. Hettiarachchi *et al.* reported that while DNA retains its B-form at neutral pH, it shifts towards the Z-form under acidic conditions (pH 3–5), driven by electrostatic attraction between the phosphate backbone and positively charged surfaces [35]. Protonation of adenine and cytosine further alters hydrogen-bonding patterns, enhancing flexibility and promoting Z-DNA stabilization. In addition, it was observed that an oligonucleotide that formed an anti-parallel G4 under acidic pH conditions switched to a double-stranded structure under neutral or alkaline pH [36]. Together, these findings suggest that acidic extracellular niches, common in biofilms and inflamed tissues, may tip the dynamic equilibrium towards the formation and stabilization of more noncanonical DNA conformations, thereby influencing the structural dynamics of extracellular DNA. Beyond these factors, extracellular and membrane-bound lipids may also modulate DNA topology. For example, high concentrations of phosphatidylethanolamine can destabilize B-DNA, whereas lower levels stabilize duplexes, suggesting a concentration-dependent influence on nucleic acid structure [37]. *In vitro* lipid modifications have likewise been shown to alter folding and stability of synthetic G4s [38]. Therefore, lipid–DNA interactions arising during membrane disruption or vesicle release may transiently alter local hydrophobicity and thereby modulate the stability or conformation of eDNA, an underexplored dimension of eDNA regulation. Consistent with this notion, G4 structures were observed in close proximity to the cell surface in *Staphylococcus epidermidis* biofilms, raising the possibility that membrane-associated components could influence eDNA conformations [39].

### Noncanonical secondary structures in biofilm eDNA

Biofilms are structured microbial communities embedded within an extracellular polymeric substance (EPS), a matrix composed of carbohydrates, lipids, proteins, and eDNA, with considerable compositional heterogeneity across species [12, 40]. Notably, eDNA is one of the few matrix components conserved across genera, emphasizing its foundational role in biofilm architecture. Although eDNA typically constitutes up to 50% of the biofilm matrix by mass depending on the species [41, 42], it plays a disproportionately critical role in biofilm architecture and function. DNA has been recognized as a component of bacterial EPS since the 1950s [43]. In 2002, eDNA was demonstrated to be an essential component of *Pseudomonas aeruginosa* biofilms [13], and since then, it has been recognized as a ubiquitous component of biofilms of diverse bacterial species and fungi [12, 44, 45]. The source of eDNA in biofilms is multifactorial. Mechanisms include active secretion of genomic DNA, often mediated by type IV and VI secretion systems; membrane vesicles and virulence factors [46] in response to quorum sensing signals [40, 47, 48]; passive release via autolysis or allolysis (fratricide) [49], antibiotic treatment [50], and the effect of monovalent cations [51]; competitive killing through toxin release, such as colicins or T6SS effectors, which lyse weaker competitors within the community [52, 53]; phage-induced lysis, including stress-activated prophage-mediated lysis, an efficient programmed route for eDNA increasing through sister-cell killing [54–57]; and incorporation of host-derived DNA such as NETs [58, 59]. Beyond its structural role as a scaffold facilitating cell attachment and aggregation, eDNA enhances biofilm mechanical integrity by forming higher-order conformations and its resilience by resisting degradation [15, 16, 60] (Fig. 1B). For instance, *Aspergillus fumigatus* biofilms rely on eDNA release as an antifungal resistance mechanism, particularly in mature communities [45]. eDNA also contributes to functional compartmentalization within the matrix, forming charged microenvironments [61] and amyloid fiber-modulated layers that modulate diffusion and signalling [62]. Functionally, eDNA acts as a reservoir for horizontal gene transfer, promoting genetic diversity and DNA damage repair [63]. It also participates in EET pathways, playing a role in energy metabolism, thereby facilitating metabolic health for bacteria in the anoxic environments of the biofilm [64]. Additionally, eDNA serves as a source of carbon, nitrogen, and phosphate [65], supporting biofilm’s survival in hostile environments [22]. Emerging evidence suggests prophage-mediated replication and extracellular synthesis of DNA within the biofilm, thereby contributing to its architecture and further highlighting the potential for other molecular processes within the extracellular milieu biofilm [66].

Recent studies have identified noncanonical secondary structures within the eDNA of biofilms, functionally distinct from their intracellular counterparts. G4s were first detected in *P. aeruginosa* biofilms under both static and dynamic conditions [15] and have since been observed in diverse species [39]. These structures could enhance the physical robustness of biofilms by assembling into higher-order networks, increasing mechanical strength, viscoelasticity, and resistance of the scaffold to nuclease digestion, particularly DNase I [15, 39], and enhancing resilience against host immunity. Moreover, once formed, G4s are thermodynamically and chemically stable, especially under physiological ionic concentrations [8], which could enable their maintenance under fluctuating environmental conditions such as oxidative stress, pH fluctuation, and variable ionic compositions.

In addition to structural roles, extracellular G4s may also function as dynamic environmental sensors that modulate biofilm physiology in response to external cues. G4 structures are known to adopt diverse intra- and intermolecular configurations and topologies—parallel, antiparallel, and hybrid forms—in reaction to local ionic strength, pH, oxidative stress, and presence of small molecules or ligands [5, 9, 19]. These structural polymorphisms, with different inherent properties, could allow G4s to respond rapidly and reversibly to environmental perturbations. G4s can bind a wide variety of small molecules, with over 4563 known ligands [67]. One such ligand is hemin, an iron-containing porphyrin derived from heme, which can accumulate in host-associated biofilms due to inflammation [11], where increased release of NETs releases endogenous hemin. Hemin-bound G4s (G4/hemin) can form catalytic, peroxidase-like DNAzymes [24]. The G4/hemin complex can function as a redox-active sensor capable of detecting external stimuli and catalysing the production of reactive oxygen species (ROS), which in turn activate various oxidative stress response pathways [68, 69]. In the biofilm milieu, this system may help promote adaptation to oxidative stress by modulating redox signalling cascades and facilitating detoxification of ROS, thereby contributing to biofilm resilience. Interestingly, however, the DNAzyme activity in biofilms seems dominated by parallel G4s with representation from RNA G4s [39]. Similarly, interaction of G4s with redox-active metabolites such as phenazine may play a role in sensing and maintaining redox balance under anoxic conditions [64, 70, 71]. In addition to sensing, G4s may also function as electrical signal conductors, facilitating EET (Fig. 1C). G4 is more electroactive than B-DNA and can transmit charges over long distances [72, 73]. In *S. epidermidis*, G4/hemin was observed to mediate EET under anoxic conditions [71]. Recent work has further suggested that noncanonical nucleic acid structures, including RNA G4s, may also contribute to EET processes in microbial systems, highlighting a broader role for nucleic acid conformations in biofilm redox biology [71, 74]. While direct evidence remains limited, G4–ligand complexes could enable biofilms to conduct electrical signals, potentially supporting metabolic cooperation and redox balance within a stratified biofilm environment.

Z-DNA, another noncanonical DNA conformation, has also been detected in microbial eDNA directly from fabric and from that of mature biofilms (grown for >24 h *in vitro*) [16, 39, 75, 76]. As described previously, Z-DNA formation is typically induced by negative supercoiling during transcription or by mechanical strain and occurs preferentially at specific repeat sequences that are widely distributed through eukaryotic and prokaryotic genomes. While Z-DNA can form at any sequence of B-DNA, poly(dG-dC) tracts have a lower energetic threshold for the B-to-Z transition than other sequences [10]. In prokaryotes, these sequences can also be found in plasmid DNA, and while long poly(dG-dC) tracts more preferentially form Z-DNA, shorter repeats may exist in a dynamic equilibrium with B-DNA [77]. Z-DNA contributes to structural integrity and nuclease resistance of biofilm eDNA [16] (Fig. 1B). While Z-DNA can be induced *in vitro* under various conditions like high salt [78], polycations [79], and oxidative modifications [80], its stability may require stabilizing proteins, with the best characterized examples being the bacterial DNA binding proteins (DNABII) [16]. These bacterial proteins localize at junction points within extracellular DNA lattice to crosslink DNA strands and confer mechanical robustness [16]. Clinical and experimental evidence highlight DNABII proteins as indispensable for biofilm maintenance: elevated DNABII expression has been reported in paediatric patient biofilm, contributing to its persistence, and antibodies targeting DNABII effectively disrupt implant-associated biofilms [81, 82]. Depletion of DNABII results in destabilization of biofilm [16]. Additionally, previous studies have revealed that HJs serve as crucial structural scaffolds for DNABII binding [83, 84]. The inability to restore biofilm integrity after DNABII depletion, even with the addition of nonspecific DNA-binding proteins, further emphasizes the specificity of DNABII–HJ interactions in matrix stability. Building on this, Buzzo *et al.* proposed a model in which DNABII proteins first stabilize HJs, generating torsional strain and closed DNA loops that favour B-to-Z transitions under supercoiling [16]. Indeed, co-crystal structures of DNABII proteins–DNA complexes show terminal nucleotides adopting anti-syn conformations characteristic of Z-DNA, supporting the idea that DNABII-stabilized HJs are critical to promote Z-DNA accumulation within the extracellular matrix. Interestingly, despite hemin’s specificity for G4s [11, 85], one study even reported that Z-DNA formation in the eDNA of *S. epidermidis* was abrogated in the absence of hemin [39]. In this system, G4s formed more readily than Z-DNA, raising the possibility that G4/hemin DNAzymes may facilitate Z-DNA nucleation, potentially through oxidative modifications such as 8-oxo-guanine formation, particularly in host-associated biofilms exposed to hemin and H_2_O_2_ during inflammation [16, 39, 86, 87]. However, Z-DNA has also been identified in contexts such as oral biofilms where free hemin is unlikely to be abundant, suggesting that additional, hemin-independent pathways likely contribute to its stabilization in microbial communities [75, 88].

### Noncanonical secondary structures in NETs eDNA

Since their discovery in 2004, the lytic and vital pathways of NETosis, their compositions, and roles in responding to an invading pathogen have been extensively studied [14]. Despite the similarities between biofilms and NETs, the existence and roles of noncanonical secondary structures like G4s and Z-DNA remain largely unexplored [14, 89]. This is particularly interesting since >70 000 potential G4s and 391 sequence-prone Z-DNA-forming sites have been detected in the human genome, and NETs are formed in conditions of high salt or oxidative stress, among others, both of which are typical of inflammation and are demonstrated to induce or stabilize G4s and Z-DNA *in vitro* [80, 90–99].

We recently discovered G4 structures in NETs, exhibiting DNAzyme activity when complexed with hemin, producing ROS that promotes bactericidal effects (Fig. 1A). Inhibiting the DNAzyme with G4-specific ligands like BRACO19 and NMM or antioxidants such as vitamin C significantly abrogated pathogen killing [17]. This highlights the critical role of G4 in NETs with potent bactericidal activity. Conversely, G4s may also protect against oxidative degradation of the eDNA by the generated hydroxyl radicals through structural shielding and interaction with DNA repair enzymes [100, 101]. G4/hemin DNAzyme in NETs emerging as the critical driver of ROS potentiates other novel roles of G4s in the system, given that comparable functions have been discovered in biofilms.

To date, reported evidence for naturally occurring Z-DNA in NETs eDNA is absent, and Z-DNA from microbial DNA [102, 103] is implicated in eliciting immune responses in the host. NETs are an important host defence mechanism against biofilm-related infections. Interestingly, NETs eDNA can be converted from B-DNA to Z-DNA when stabilized by bacterial DNABII proteins, which offers adaptive benefits for biofilm to incorporate NETs eDNA into its EPS [16, 81] (Fig. 1B). Recent clinical evidence further suggests elevated DNABII expression in biofilms from paediatric patients, underscoring its relevance *in vivo* [81]. This reinforces biofilm’s structural integrity and effectively creates a protective niche against NETs killing by hijacking the host immune mechanism for its own benefit [16, 39]. It also highlights the propensity for Z-DNA formation in NETs eDNA under the right conditions, which may enhance the stability, protection, and persistence of NETs eDNA, thereby sustaining immune defence mechanisms against biofilms. For instance, such properties contribute to the formation of a ‘dead zone’ in the eye, effectively confining *P. aeruginosa* biofilms and preventing their dissemination to the brain [104]. A secondary defence mechanism by biofilms against NETs is the release of the bacterial nucleoid-associated protein, H-NS, as the eDNA matures from B- to Z-DNA, which not only prevents the formation of new NETs but also leads to the retraction of previously formed NETs, offering a unique edge to the spread of the pathogen [105].

### Can Z-DNA naturally occur in NETs?

The process of NETosis, driven by oxidative stress and chromatin decondensation, creates an environment that is conducive for the formation of structures like G4 and Z-DNA through the formation of 8-oxo-guanine (8-oxoG) [80, 97–99]. These oxidative nucleotides, intracellularly repaired by the BER pathway primarily through the activity of 8-oxoguanine DNA glycosylase 1 (OGG1), are excised, creating abasic sites that serve as formation points for secondary structures like G4 and Z-DNA [106, 107]. Interestingly, the inhibition of early steps in BER, particularly OGG1 activity, suppresses neutrophil infiltration and spontaneous NETosis, suggesting NETosis and the pathway to the formation of noncanonical secondary structures are interconnected [7, 108]. Furthermore, Z-DNA formation has been implicated in chromatin remodelling during transcriptional activation by disruption and ejection of nucleosomes to maintain an open chromatin structure [109]. This phenomenon is particularly relevant in the context of NETosis, where chromatin decondensation and nucleosome eviction are fundamental processes enabling the release of nuclear DNA [110]. When histones dissociate and the DNA ends remain topologically constrained, regions of negative supercoiling may arise, creating favourable conditions for B-to-Z transition. Oxidative lesions may further promote this structural conversion. Additionally, mitochondrial DNA (mtDNA), released extracellularly alongside nuclear DNA during NETosis, has a closed circular genome rich with unmethylated CpG from its bacterial ancestry and is highly prone to torsional stress from supercoiling and oxidative damage, both favouring transition from B- to Z-DNA [111–114]. In addition, NETs must also function in DNase-rich blood (up to 0.356 U/ml in healthy individuals), where nuclease resistance is essential, and Z-DNA is ∼12% longer than B-DNA per bp and ∼37% longer per turn, potentially casting a wider net to enhance pathogen entrapment, presenting a favourable scenario for the existence of Z-DNA in NETs [115, 116], through stabilized nuclear aggregates of polyamines hypothesized in 2013 [117]. While the presence of these noncanonical secondary structures proves advantageous to the overall functioning of NETs, persistence of NETs can exacerbate inflammation, hinder NET clearance, and contribute to chronic diseases [118, 119]. Understanding the balance between their protective roles and pathological consequences remains essential for addressing NET-related immunopathologies.

### eDNA structures contributing to immune modulation and pathologies

DNA is well-recognized as PAMPs or DAMPs, depending on its origin, structural features, or context of exposure. As a PAMP, DNA is typically derived from a microbial source, often containing unmethylated CpG motifs, along with other microbial components, marking it as foreign to the host immune system [120]. Conversely, DAMP DNA originates from the host itself, released during cell stress, injury, or death [121]. Contextual cues, including sequence features, ectopic location, chemical modifications, molecular complexes, or associated proteins, can render self-DNA immunogenic. PRRs, most notably Toll-like receptor 9, cyclic GMP-AMP synthase (cGAS), absent in melanoma 2 (AIM2), and ZBP-1, are endosomal or cytosolic immune sensors that can recognize these DNA signatures, leading to activation of type 1 interferons (IFN), inflammasomes, and necroptosis. However, immune recognition of eDNA would first require its internalization, via natural endocytosis or forming complexes with antibodies or DNA-binding proteins, into host cells before being sensed by PRRs.

Biofilm eDNA can act as a PAMP and trigger host immune responses. Endosomal TLR9 in macrophages and gingival fibroblasts was activated by periodontal biofilm eDNA, inducing inflammatory mediators and contributing to tissue degradation [122]. *Pseudomonas aeruginosa* biofilm eDNA could activate neutrophils marked by cytokine release, upregulation of activation markers, and enhanced phagocytosis and NETosis [104]. eDNA degradation by DNase I significantly attenuates these immune responses, reinforcing eDNA’s role as a key PAMP [123]. Interestingly, this neutrophil activation appears to occur independently of CpG and TLR9 signalling, suggesting alternative DNA sensors are involved. Although direct evidence remains lacking, possible candidates might include cGAS-STING–associated DNA receptors or other pattern recognition receptors yet to be characterized. Notably, recent reports have described the existence of cell surface TLR9 (sTLR9), which can bind CpG oligodeoxynucleotides; however, whether sTLR9 can directly initiate signal transduction remains uncertain. sTLR9 expression has been documented on neutrophils, B cells, and erythrocytes, raising the intriguing possibility that surface-localized DNA recognition contributes to biofilm eDNA sensing *in vivo* [124, 125].

Circumstantial evidence implicates noncanonical structures of biofilm’s eDNA as PAMPs. Z-DNA is an effective immunogen that can potently induce antibodies by immunization under conditions in which B-DNA is inactive [126]. Interestingly, Z-DNA-specific antibodies—thought to originate from potential biofilm exposure—have been detected not only in patients with autoimmune diseases such as rheumatoid arthritis (RA) and systemic lupus erythematosus (SLE), but also in the sera from healthy individuals [102]. In addition to antibody recognition, host recognition of extracellular Z-DNA is likely a key event, with Z-DNA binding protein 1 (ZBP1) representing a potential host innate immune sensor candidate. Although direct recognition of biofilm’s extracellular Z-DNA by host ZBP1 remains to be confirmed *in vivo*, ZBP1 plays a pivotal role in inducing inflammatory cell death and type I IFN responses during viral infections [127]. The presence of Z-DNA or Z-DNA–binding proteins in fungi has not yet been fully established; however, ZBP1 can detect fungal nucleic acids and promote inflammasome activation and PANoptosis, suggesting a potential link between fungal eDNA and Z-DNA–mediated immune recognition [128]. Similarly, despite the lack of direct evidence supporting G4 as a PAMP in biofilm eDNA, G4 in an unmethylated CpG oligomer can increase immunostimulation through activation of TLR9, which can bind and recognize G4 structures [129].

G4 and Z-DNA in biofilm’s eDNA can also modulate immune signalling. The physical properties of G4 and Z-DNA render them resistant to host nucleases, allowing biofilms to persist despite immune pressure [16, 39]. Chloroquine, a DNA intercalator that preferentially binds to B-DNA, has also been shown to bind to Z-DNA, reverting it to B-DNA, and restore DNase susceptibility and reduce biofilm mass [16, 75, 130, 131]. Conversely, the B- to Z-DNA transition mediated by DNABII enables mature biofilms to co-opt host NETs as protective barriers rather than immune effectors. At the same time, this conversion disrupts NET antimicrobial activity by displacing histones and neutralizing their bactericidal potential [16]. G4-eDNA, by forming higher-order biofilm structure, can not only shield the underlying bacterial PAMPs from recognition by PRRs, but G4s can also bind and sequester proinflammatory host molecules like hemin [11].

NETs are potent DAMPs that play a dual role in antimicrobial defence and immune modulation [132]. While NETs effectively trap and eliminate pathogens, excessive NET formation or reduced clearance can lead to tissue damage and systemic inflammation. This can be mediated by the release of histones, ROS, and proteases, as well as the eDNA itself, engaging in multiple arms of the innate immune system [132, 133]. NETs eDNA is known to trigger various proinflammatory cytokines via TLR9 or cGAS in immune cells. Though unconfirmed by direct evidence in the context of NETosis, mtDNA released from mitochondria into cytoplasm or extracellularly under oxidative or genotoxic stress in various biological contexts has been shown to adopt Z-conformation and serve as a structural DAMP, engaging ZBP-1 and cGAS, which can promote proinflammatory responses and necroptotic cell death [134]. For instance, in keratinocytes exposed to ultraviolet radiation, the accumulation of mitochondrial-derived Z-DNA correlates with heightened Type 1 IFN production compared to B-DNA. This immune response, however, is mitigated when ZBP-1 is knocked down, indicating that ZBP1 functions as the sensor mediating IFN induction downstream of Z-DNA recognition. Moreover, the G4/hemin DNAzyme in NETs eDNA was shown to be the primary producer of the hydroxyl radical, the most potent ROS, which indiscriminately causes oxidative damage and can lead to haemolysis of red blood cells or other tissues in the vicinity [17]. Another critical factor contributing to imbalance is the accumulation of DNA–histone complexes and protein-associated DNA in the extracellular space due to insufficient DNase activity [119, 135]. Under normal conditions, DNase I degrades naked B-DNA, while DNase1L3 specifically targets protein-bound DNA in NETs, preventing excessive immune activation [136, 137]. However, G4s, and potentially Z-DNA, in NETs can enhance their resistance to degradation, prolonging their immune engagement [16, 39] (Fig. 1B). In fact, the presence of a nuclease-resistant G4 showed a 10-fold increase in the uptake of a CpG oligonucleotide by human B lymphocyte cell lines, resulting in more than double the amount of IL-6 produced [138]. This resistance to degradation may worsen systemic inflammation, tissue damage, and organ dysfunction and drive autoimmune diseases.

NETs and their impaired clearance have been implicated in autoimmune conditions like SLE through multiple lines of evidence. Autoantibodies against nucleic acids and excessive type I IFN are hallmarks of SLE. Internalization of SLE immune complexes containing nucleic acids by plasmacytoid dendritic cells (pDCs) induces endosomal TLR9 activation and type I IFN production [139]. Besides double-stranded DNA, SLE autoantibodies also recognize other components of NETs, such as histones, antimicrobial peptides, MPO, and proteinase 3, suggesting the direct role for NETs in the initiation and perpetuation of SLE via the generation of autoantigens [140, 141]. Specific posttranslational modifications of histones on NETs have already been identified as immunogens of SLE. Hydroxyl radicals, likely including those generated by the G4/hemin DNAzyme of NETs eDNA, can give rise to various posttranslational modifications like hydroxylation, sulfoxidation, and carbonylation of NET-associated proteins that could become immunogenic [142]. In addition, antigenic complexes made up of NET-derived eDNA and antimicrobial peptides like LL-37 have been shown to reduce NET clearance and drive chronic inflammation through the activation of pDCs and autoantibody production via memory B cells in SLE patients [143, 144]. Interestingly, LL-37, a cationic amphipathic antimicrobial peptide from the cathelicidin family, can potentially form an antigenic complex with Z-DNA both through charge as well as its alpha helical structure. Moreover, neutrophils from SLE patients, particularly low-density granulocytes, are prone to produce more NETs with enriched oxidized eDNA, which can not only impair nuclease degradation but also increase their sensing by the cGAS pathway, further augmenting interferongenic responses [145, 146]. As noted previously, oxidized nucleic acid is prone to B-to-Z transformation. Besides excess formation of NETs, many SLE patients have impaired ability to degrade these structures due to aberrant levels or functions of DNases [147]. Additionally, ZBP-1, a key inducer of type 1 IFN response, has been found to be significantly upregulated in patients with SLE compared to healthy individuals [148]. Along with expression of autoantibodies against DNA, patients with SLE—and even healthy subjects—express anti-Z-DNA antibodies, which, to date, are thought to originate in response to extracellular Z-DNA from biofilms [102, 149]. Given the conjectural evidence presented above and the prevalence of anti-Z-DNA antibodies in healthy individuals, we propose that NET-derived Z-DNA is an endogenous source of autoantigens, which may play a central role in SLE pathogenesis. Additional research is needed to clarify the mechanisms underlying immune tolerance to these autoantigens in healthy individuals and how disruptions in these processes contribute to the development of autoimmunity like SLE.

In addition to being proinflammatory, NETs have also been implicated in being prothrombotic, with elevated NET levels in COVID-19 patients correlating with heightened thrombotic events [150, 151]. Specifically, since the NETs eDNA is anionic, it activates the contact pathway and has been shown to directly interact with thrombin, especially at exosite II [152, 153]. While direct evidence is absent, a mixed duplex and G4-forming aptamer, HD22, showed specificity for exosite II with potential allosteric activation of thrombin and procoagulant effects on fibrin formation, highlighting a potential role for noncanonical secondary structures in NETs eDNA regulating thrombosis, further emphasizing the need for directed investigations [154, 155].

### Targeting noncanonical secondary structures: potential therapeutic implications and beyond

G4s and Z-DNA in the eDNA confer enhanced stability, immune evasion or dysregulation, and resistance to nuclease-mediated degradation in both biofilms and NETs, contributing to chronic infection, persistent inflammation, and autoimmune pathology. Targeting these noncanonical structures offers a promising strategy to disrupt pathological biofilm integrity and limit excessive NET accumulation without significantly impairing antimicrobial defences.

One strategy to disrupt biofilm is to leverage G4 DNAzymes. Inducing peroxidase activity within biofilms via G4/hemin can generate ROS, effectively kill bacteria, and disrupt biofilms [17, 39, 156]. This effect may be potentiated with DNAzyme enhancers like spermine or chitosan, as well as G4 stabilizers, particularly when combined with antibiotics that are tolerant to H_2_O_2_ to improve biofilm penetration and achieve synergistic outcomes [27, 157, 158]. Conversely, spermine may stabilize Z-DNA, strengthening the biofilm matrix; hence, further experimental validation is required to determine whether DNAzyme potentiation or Z-DNA stabilization is facilitated by spermine treatment [33]. A study utilizing G4 hydrogel as a potential nonantibiotic wound dressing for biofilm infections demonstrated that the supply of hemin and H₂O₂ from the hydrogel activated ROS mediated by G4 rapidly eradicated biofilm infections and improved healing in a diabetic wound mouse model [156].

Instead of exploiting the enzymatic activity of G4 in biofilms, an alternative approach is to destabilize the G4 structure, rendering the extracellular DNA more susceptible to nuclease digestion. Small molecules, such as TMPyP4, a porphyrin-based molecule known to intercalate into G4-DNA, can disrupt G4 when used at specific concentrations that favour unwinding rather than stabilization [159–161]. Antisense oligonucleotides, complementary to the G4-forming strand, can also be strategically designed and have been shown to unwind G4 structures found in telomeres and promoters [162, 163]. Although direct evidence in biofilm is limited, helicases are also known to unwind G4 structures [164, 165]. Examples include members of the RecQ, XPD, Pif1, and DEAH box family helicases [166, 167]. Fracchioni *et al.* recently provided an in-depth review of emerging strategies for selectively unfolding G4s, highlighting their potential to modulate G4 biological functions and enhance therapeutic applications [163].

Given Z-DNA’s role in providing stability to biofilms, therapies targeting Z-DNA directly and its stabilizing proteins aim to disrupt its secondary structure, ultimately inducing collapse. An emerging therapeutic that targets Z-DNA is chloroquine, an antimalarial and immunosuppressant drug that is commonly used for treating disorders like SLE [130, 168]. Chloroquine stabilizes DNA in its B-form, effectively disrupting Z-DNA-dependent biofilm stability by inhibiting the B-to-Z DNA transition [169]. By preferentially binding to Z-DNA-forming sequences, chloroquine reduces Z-DNA content and mature biofilm mass, shifting bacteria to their planktonic state and restoring mature biofilm’s susceptibility to DNase [16]. Interestingly, mechanical testing showed that chloroquine-treated biofilms had significantly lowered stiffness, indicating disrupted biofilm EPS, whereas DNase treatment increased stiffness by depleting B-DNA, consistent with the notion that a greater proportion of B-DNA contributes to a more flexible biofilm matrix. A recent *Streptococcus mutans* biofilm study showed that eDNA resistant to DNase I could be effectively degraded when DNase I was combined with chloroquine, emphasizing the translational potential of targeting noncanonical DNA structures in biofilm [75]. In addition, Holliday junction resolvase RusA, which cleaves crossover DNA structures, has demonstrated potential for Z-DNA elimination, owing likely to the dependence on HJs for the stabilization of vertices by DNABII and formation of Z-DNA in the biofilm [16, 170]. Additionally, due to the thermodynamically unstable nature of Z-DNA, stabilizer proteins (DNABII), such as HU and IHF, stabilize Z-DNA within biofilms and provide promising targets for indirectly collapsing Z-DNA in biofilms [171]. Targeting these proteins with monoclonal antibodies (e.g. CMTX-101, TRL1068) destabilizes Z-DNA, collapsing biofilms and restoring antibiotic susceptibility [171, 172]. NETs, unlike biofilms, form an integral part of the immune response to trap and kill pathogens and should not be abrogated. Current therapies, such as anti-TNF treatments, often target neutrophil function but carry risks like neutropenia and increased susceptibility to infections [173, 174]. Completely inhibiting NETosis can also weaken innate immune defences, as seen in cases like ocular *P. aeruginosa* biofilm infections, where NET inhibition led to bacterial migration and worsened outcomes [104]. Given this challenge, therapies should prioritize enhancing NET clearance over complete suppression; hence, we propose targeting the secondary structures of NETs eDNA to reduce undue effects from their excessive accumulation. These structures may be destabilized by modulating environmental factors such as ionic strength and oxidative stress, or via engineered nucleases specifically targeting G4 or Z-DNA [80, 99, 175, 176]. Alternatively, the degradation of Z-DNA may be influenced by the activity of DNA unwinding enzymes, particularly topoisomerases. Topoisomerases, known for their ability to resolve DNA supercoiling, indirectly modulate Z-DNA stability by relaxing negative superhelical stresses in B-DNA. This relaxation would prevent DNA from shifting into the Z-form, thereby reducing the presence of Z-DNA structures and rendering NET-derived eDNA less resistant to DNase degradation [177]. Combining these approaches—targeted nucleases, DNA unwinding strategies, and environmental modulation—presents a unified framework for mitigating NET-driven inflammation. By preserving NETs' protective functions while preventing their pathological effects, these strategies could lead to more precise and effective treatments for a wide range of immune disorders.

## Conclusions and future research

Despite limited but growing evidence, Z-DNA and G4 structures remain the best-characterized noncanonical conformations in extracellular DNA of biofilms and NETs. Nonetheless, several questions arise about these noncanonical structures in the two systems (Panel). Interestingly, while G4s present in NETs eDNA play a major role in killing invading pathogens; however, when complexed with hemin, the same complex in biofilm eDNA works towards detoxifying ROS and facilitating EET when anoxic. The contextual evolution of the same DNAzyme in antithetical systems suggests a less understood ‘arms race’. Additionally, discovering Z-DNA in NETs eDNA offers a paradigm shift in the understanding of the immunopathogenesis of SLE. It explains finding anti-Z-DNA antibodies in healthy individuals and questions biofilm eDNA as the antigen; it also warrants further studies into the body’s tolerance mechanisms towards its own highly immunostimulatory Z-DNA. Finally, given the immunomodulatory roles these structures play, it is of utmost importance to target these structures specifically to facilitate rapid clearance of these eDNA systems. Hence, G4 and Z-DNA likely act as a double-edged sword whose effects are delicately balanced by timely clearance of the eDNA in these systems.

## Panel

### Outstanding questions

1. Can stable non-canonical secondary structures be formed in the eDNA of biofilms and NETs?
  - What are the shared structures between the two systems, and do they have similar or divergent functions in each system?
  - What are the key factors that stabilize these alternative structures in each system?
2. Do non-canonical structures have novel roles in the eDNA that are distinct from their intended intracellular roles?
  - Are the non-canonical secondary structures the underlying engine providing the bioactivity to the eDNA in biofilms and NETs?
  - Are there variations in the numbers of the non-canonical secondary structures formed in the eDNA dependent on environmental cues?
  - Do they adapt functionally in response to perturbations in the environment?
3. How do non-canonical secondary structures in eDNA facilitate or mitigate hostile interactions between NETs and biofilms?
  - Is there structural complementarity between non-canonical eDNA structures in NETs and biofilms that enables physical co-assembly or cross-organization of their matrices?
  - Could mixed DNA structures interfere with innate immune sensing, leading to immune tolerance or inappropriate activation?
4. Can non-canonical secondary structures of these systems act as effectors of immunomodulation?
  - Can naked non-canonical secondary structures act as DAMPs or PAMPs?
  - Are circumstances and contextual cues important for recognition and internalization of these non-canonical secondary structures?
5. Do non-canonical structures in the eDNA of NETs play a role in immunopathologies?
  - Are non-B structures more immunostimulatory? What is the mechanism of tolerance to self-non-B structures in eDNA of NETs?
  - What are their roles in thrombus formation?
6. Can targeting these core non-canonical structures offer novel therapeutic modalities to treat biofilm- and NET-related pathologies?
  - Can leveraging versus destroying the non-canonical secondary structures in the biofilm eDNA offer an antibiotic synergistic/free strategy to disrupt biofilms?
  - How can NETs be cleared by neutralizing the non-canonical secondary structures without compromising their immune defensive response?

## Data Availability

No new data were generated or analysed in support of this research.
